# Silicon quantum processor with robust long-distance qubit couplings

**DOI:** 10.1038/s41467-017-00378-x

**Published:** 2017-09-06

**Authors:** Guilherme Tosi, Fahd A. Mohiyaddin, Vivien Schmitt, Stefanie Tenberg, Rajib Rahman, Gerhard Klimeck, Andrea Morello

**Affiliations:** 1 Centre for Quantum Computation and Communication Technology, School of Electrical Engineering & Telecommunications, UNSW, Sydney, NSW 2052 Australia; 2 Network for Computational Nanotechnology, Purdue University, West Lafayette, IN 47907 USA; 3 Present Address: Quantum Computing Institute, Oak Ridge National Laboratory, Oak Ridge, 37830 TN USA

## Abstract

Practical quantum computers require a large network of highly coherent qubits, interconnected in a design robust against errors. Donor spins in silicon provide state-of-the-art coherence and quantum gate fidelities, in a platform adapted from industrial semiconductor processing. Here we present a scalable design for a silicon quantum processor that does not require precise donor placement and leaves ample space for the routing of interconnects and readout devices. We introduce the flip-flop qubit, a combination of the electron-nuclear spin states of a phosphorus donor that can be controlled by microwave electric fields. Two-qubit gates exploit a second-order electric dipole-dipole interaction, allowing selective coupling beyond the nearest-neighbor, at separations of hundreds of nanometers, while microwave resonators can extend the entanglement to macroscopic distances. We predict gate fidelities within fault-tolerance thresholds using realistic noise models. This design provides a realizable blueprint for scalable spin-based quantum computers in silicon.

## Abstract (web summary)

Quantum computers will require a large network of coherent qubits, connected in a noise-resilient way. Tosi et al. present a design for a quantum processor based on electron-nuclear spins in silicon, with electrical control and coupling schemes that simplify qubit fabrication and operation.

## Introduction

The successful implementation of quantum algorithms requires incorporation of error correction codes^1^ that deal with the fragile nature of qubits. The highest tolerances in error rates are found when using nearest-neighbor topological codes^2^, long-distance entanglement links^3^, or a combination of both^4^. There exist several physical platforms where state preservation^5–7^, qubit control^8–11^, and two-qubit logic gates^8, 12^ are achieved with fault-tolerant fidelities. The ultimate goal is to integrate a large number of qubits in expandable arrays to construct a scalable, universal quantum processor.

Donor spin qubits in silicon are an appealing physical platform for that goal, due to their integrability with metal-oxide-semiconductor (MOS) structure and nanometric unit size^13^. By using isotopically enriched ^28^Si as the substrate material^14^, donor spins offer coherence times around a second (for the electron) or a minute (for the nucleus)^7^, up to hours in bulk ensembles^6^, and control error rates as small as 10^−4^ ^11^. However, integrating several of these qubits in a scalable architecture remains a formidable challenge, mainly because of the difficulty in achieving reliable two-qubit gates.

The seminal Kane proposal^15^ for a nuclear-spin quantum computer in silicon described the use of short-range exchange interactions *J* between donor-bound electrons, to mediate an effective inter-nuclear coupling of order ~100 kHz at a ~15 nm distance. However, the exchange interaction has an exponential and oscillatory spatial behavior that can result in an order of magnitude variation in strength upon displacement by a single lattice site^16, 17^. Notwithstanding, plenty of progress has been made in the experimental demonstration of the building blocks of a Kane-type processor^18–21^, including the observation of inter-donor exchange^22–24^. Slightly relaxed requirements on donor placement can be found when using a hyperfine-controlled exchange interaction between electron spin qubits^25^, or a slower magnetic dipole-dipole coupling effective at ~30 nm distances^26^. Other proposals space donors further apart by introducing some intermediate coupler, e.g., donor chains^27, 28^, charge-coupled devices^29^, ferromagnets^30^, probe spins^31^, or quantum dots^32^.

Here we introduce the design of a large-scale, donor-based silicon quantum processor based upon electric dipole interactions. This processor could be fabricated using existing technology, since it does not require precise donor placement. The large inter-qubit spacing, >150 nm, leaves sufficient space to intersperse classical control and readout devices, while retaining some of the compactness of atomic-size qubits. Stabilization schemes largely decouple the qubits from electric noise while still keeping them sensitive to electric drive and mutual coupling. Finally, the whole structure retains the standard silicon MOS materials stack, important for ultimate manufacturability.

## Results

### Coupling Si:P spin qubits to electric fields

**Fig. 1:**
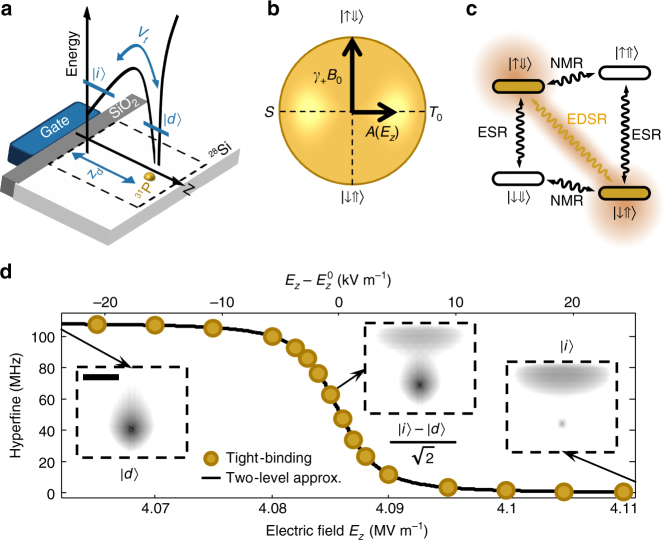
Coupling donor spin qubits to electric fields via hyperfine modulation. **a** Qubit unit cell, in which the electron interface state, |*i*〉, is coupled to the donor-bound state, |*d*〉, by a tunnel rate *V*_t_. The *solid black line* represents the conduction band profile along *z*. **b** Bloch sphere of a flip-flop spin qubit coupled to a vertical electric field *E*_z_ via the hyperfine interaction *A*. Electron-nuclear singlet and triplet states are denoted by $S = \left( {\left| { \downarrow \Uparrow } \right\rangle - \left| { \uparrow \Downarrow } \right\rangle } \right){\rm{/}}\sqrt 2$ and ${T_0} = \left( {\left| { \downarrow \Uparrow } \right\rangle + \left| { \uparrow \Downarrow } \right\rangle } \right){\rm{/}}\sqrt 2$. **c** Si:P electron-nuclear spin levels, showing standard electron spin resonance (*ESR*) and nuclear magnetic resonance (*NMR*) transitions, together with hyperfine-enabled EDSR. **d** Atomistic tight-binding simulations^72^ (*dots*) of the electron-nucleus hyperfine interaction, for a *z*_d_ = 15.2 nm deep donor, as a function of vertical electric field. The *solid line* is a fit using the simplified two-level Hamiltonian ${{\cal H}_{{\rm{orb}}}} + {\cal H}_A^{{\rm{orb}}}$, which yields *V*_t_ = 9.3 GHz (see Supplementary Note 1). The *insets* show the electron ground-state wavefunction, $\left| g \right\rangle$, in the region within *dashed lines* in **a**, for three different vertical electric fields. *Scale bar* is 10 nm

The phosphorus donor in silicon comprises an electron spin *S* = 1/2 with gyromagnetic ratio *γ*_e_ = 27.97 GHz T^−1^ and basis states $\left| \downarrow \right\rangle$, $\left| \uparrow \right\rangle$, and a nuclear spin *I* = 1/2 with gyromagnetic ratio *γ*_n_ = 17.23 MHz T^−1^ and basis states $\left| \Downarrow \right\rangle ,\left| \Uparrow \right\rangle$. The electron interacts with the nucleus through the hyperfine coupling *A* ≈ 117 MHz. When placed in a large magnetic field *B*_0_ (${\gamma _ + }{B_0} \gg A$, with *γ*_+_ = *γ*_e_ + *γ*_n_), the eigenstates of the system are the separable tensor products of the basis states, i.e., $\left| { \downarrow \Uparrow } \right\rangle$, $\left| { \downarrow \Downarrow } \right\rangle$, $\left| { \uparrow \Downarrow } \right\rangle$, $\left| { \uparrow \Uparrow } \right\rangle$ (Fig. 1c). The electron and the nucleus can be operated as single qubits by applying oscillating magnetic fields resonant with any of the transitions frequencies between eigenstates that differ by the flipping of one of the spins, e.g., $\left| { \downarrow \Uparrow } \right\rangle$ ↔ $\left| { \uparrow \Uparrow } \right\rangle$ for the electron qubit, etc. (Fig. 1c).

We envisage a device where a shallow ^31^P donor is embedded in an isotopically enriched ^28^Si crystal at a depth *z*_d_ from the interface with a thin SiO_2_ layer (Fig. 1a). The orbital wavefunction *ψ* of the donor-bound electron can be controlled by a vertical electric field *E*_*z*_ applied by a metal gate on top. It changes from a bulk-like donor state at low electric fields to an interface-like state at high fields^33, 34^ (*insets* in Fig. 1d). The hyperfine interaction *A*(*E*_*z*_), proportional to the square amplitude of the electron wavefunction at the donor site |*ψ*(0, 0, *z*_d_)|^2^, changes accordingly from the bulk value *A* ≈ 117 MHz to *A* ≈ 0 when the electron is fully displaced to the interface (Fig. 1d). Shifting the electron wavefunction also results in the creation of an electric dipole *μ*_e_ = *ed*, where *e* is the electron charge and *d* is the separation between the mean positions of the donor-bound and interface-bound wavefunctions (*d* ≲ *z*_d_, see Supplementary Note 1). The induced electric dipole *μ*_e_ has been largely overlooked in the past, but plays a crucial role in this proposal.

The key idea is to define a new qubit, called henceforth the flip-flop qubit, described in the subspace spanned by the states $\left| { \downarrow \Uparrow } \right\rangle$, $\left| { \uparrow \Downarrow } \right\rangle$. Transitions between these basis states cannot be induced by magnetic resonance, because there is no change in the *z*-component of the total angular momentum. However, the hyperfine interaction, *A* **S** ⋅ **I**, is a transverse term in the flip-flop basis, since its eigenstates are $S = \left( {\left| { \downarrow \Uparrow } \right\rangle - \left| { \uparrow \Downarrow } \right\rangle } \right){\rm{/}}\sqrt 2$ and ${T_0} = \left( {\left| { \downarrow \Uparrow } \right\rangle + \left| { \uparrow \Downarrow } \right\rangle } \right){\rm{/}}\sqrt 2$ (Fig. 1b). Therefore, electrically modulating *A*(*E*_z_) at the frequency

1 $${\epsilon _{{\rm{ff}}}}(A) = \sqrt {{{\left( {{\gamma _ + }{B_0}} \right)}^2} + {{\left[ {A\left( {{E_{\rm{z}}}} \right)} \right]}^2}} ,$$

corresponding to the flip-flop qubit energy splitting, causes an electric dipole spin resonance (EDSR) transition between the $\left| { \downarrow \Uparrow } \right\rangle$, $\left| { \uparrow \Downarrow } \right\rangle$ basis states^35, 36^ (Fig. 1c). This transition is faster at the “ionization point”, where the electron is shared halfway between donor and interface, since *A*(*E*_*z*_) can vary strongly upon the application of a small voltage on the top gate.

### Electrical noise and relaxation

Since the qubit operation is based upon the use of electric fields, a natural concern is the fragility of the qubit states in the presence of electric noise. Below we show that there are special bias points that render the flip-flop qubit operation highly robust against noise.

A quantum-mechanical description of the system is obtained by treating also the electron position as a two-level system (effectively a charge qubit; see Supplementary Note 1 for a justification of this two-level approximation), where the vertical position of the electron is represented by a Pauli *σ*_*z*_ operator, with eigenvectors |*d* 〉, for the electron at the donor, and |*i* 〉 at the interface (Fig. 1a, d). The simplified orbital Hamiltonian reads (in units of Hz):

2 $${{\cal H}_{{\rm{orb}}}} = \frac{{{V_{\rm{t}}}{\sigma _{\rm{x}}} - \left[ {e\left( {{E_{\rm{z}}} - E_{\rm{z}}^0} \right)d{\rm{/}}h} \right]{\sigma _{\rm{z}}}}}{2},$$

where *V*_t_ is the tunnel coupling between the donor and the interface potential wells, $E_{z}^0$ is the vertical electric field at the ionization point, and *h* is the Planck constant. The electron ground |*g* 〉 and excited |*e* 〉 orbital eigenstates depend on *E*_*z*_ (Fig. 1d) and have an energy difference given by:

3 $${\epsilon _{\rm{o}}} = \sqrt {{{\left( {{V_{\rm{t}}}} \right)}^2} + {{\left[ {e\left( {{E_{z}} - E_{z}^0} \right)d{\rm{/}}h} \right]}^2}}$$

**Fig. 2:**
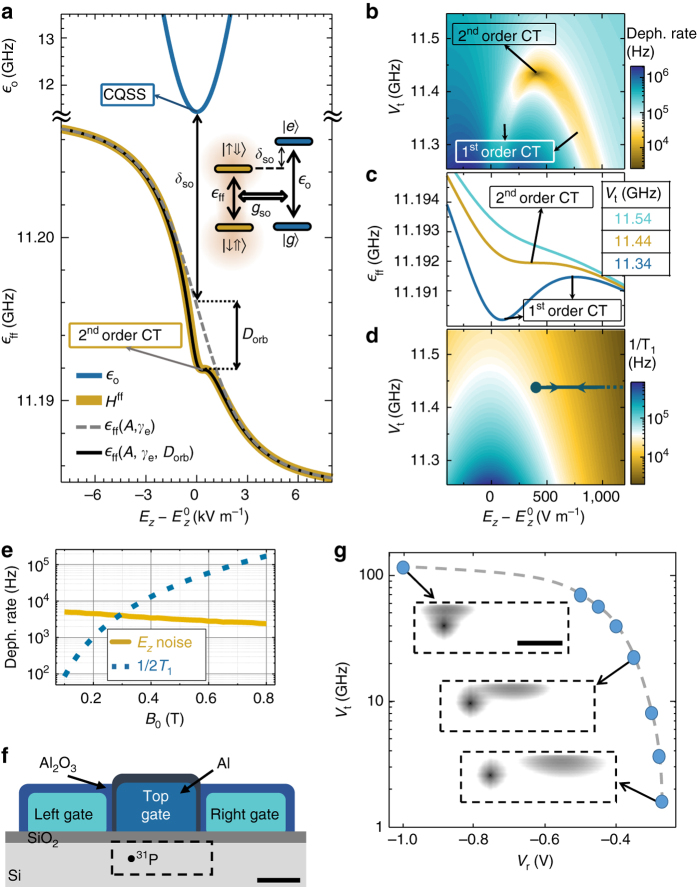
Robustness to electric noise. **a** Charge, ${\epsilon _o}$, and flip-flop, ${\epsilon _{{\rm{ff}}}}$, qubit transition frequencies as a function of vertical electric field *E*_*z*_, for *B*_0_ = 0.4 T, *A* = 117 MHz, *d* = 15 nm, Δ_γ_ = −0.2% and *V*_t_ = 11.44 GHz. The *inset* shows the level diagram of flip-flop states coupled to charge states. *CT* stands for “clock transition” and *CQSS* for “charge qubit sweet spot”. **b** Estimated flip-flop qubit dephasing rate, assuming electric field noise $E_{z{\rm{,rms}}}^{{\rm{noise}}} = 100$ V m^−1^. **c** *E*_*z*_-dependence of flip-flop precession frequency for the three indicated tunnel coupling values. **d** Flip-flop qubit relaxation rate, with *arrows* indicating the adiabatic path used for *z*-gates. **e** Flip-flop qubit dephasing rate due to *E*_z_ noise and relaxation, at second-order CTs for each *B*_0_. **f** Device structure to tune the tunnel coupling *V*_t_ of the charge qubit. *Scale bar* is 30 nm. **g** *V*_t_ as a function of right gate voltage, calculated using a finite element Poisson solver (Synopsis TCAD) and atomistic tight biding (NEMO-3D)^72^. The *insets* illustrate the NEMO-3D wavefunctions inside *dashed region* in **f**, for three right gate voltages *V*_r_ = −1, −0.35 and −0.27 V. The left gate voltage is *V*_l_ = −0.5 V for all the simulations, and the top gate is biased such that the position of the electron is in between the donor and interface. *Scale bar* is 20 nm. The donor is assumed to be *z*_d_ = 9.2 nm below the Si/SiO_2_ interface

At the ionization point, the energy difference between eigenstates $\left| e \right\rangle = \left( {\left| d \right\rangle + \left| i \right\rangle } \right){\rm{/}}\sqrt 2$ and $\left| g \right\rangle = \left( {\left| d \right\rangle - \left| i \right\rangle } \right)/\sqrt 2$ is minimum and equal to *V*_t_ (Fig. 2a), and therefore first-order insensitive to electric noise, ∂${\epsilon _o}$/∂*E*_z_ = 0. This bias point is referred to as the “charge qubit sweet spot”^37^ (CQSS—Fig. 2a).

Conversely, the bare flip-flop qubit energy is expected to depend strongly on *E*_*z*_, through the combined effect of the hyperfine interaction *A* (Eq. 1) and the orbital dependence of the electron gyromagnetic ratio, *γ*_e_. Indeed, the gyromagnetic ratio of an electron confined at a Si/SiO_2_ interface can differ from that of a donor-bound electron by a relative amount Δ_*γ*_ up to 0.7%^38^. Therefore, the Zeeman terms in the Hamiltonian must include a dependence of the electron Zeeman splitting on its orbital position, i.e., the charge qubit *σ*_*z*_ operator:

4 $${\cal H}_{{B_0}}^{{\rm{orb}}} = {\gamma _{\rm{e}}}{B_0}\left[ {1 + \left( {\frac{{1 + {\sigma _{z}}}}{2}} \right){\Delta_\gamma }} \right]{S_{\rm{z}}} - {\gamma _{\rm{n}}}{B_0}{I_{z}}.$$

We can also write the hyperfine coupling as an operator that depends on the charge qubit state:

5 $${\cal H}_A^{{\rm{orb}}} = A\left( {\frac{{1 - {\sigma _{\rm{z}}}}}{2}} \right){\bf{S}} \cdot {\bf{I}}$$

Indeed, this simple two-level approximation, shown as a *black line* in Fig. 1d, reproduces the full tight-biding simulations (*yellow dots*).

The overall flip-flop qubit transition frequency as a function of *E*_*z*_ becomes:

6 $${\epsilon _{{\rm{ff}}}}\left( {A,{\gamma _{\rm{e}}}} \right) = \sqrt {{{\left[ {{\gamma _{\rm{e}}}\left( {{E_{\rm{z}}}} \right) + {\gamma _{\rm{n}}}} \right]}^2}{B_0}^2 + {{\left[ {A\left( {{E_{\rm{z}}}} \right)} \right]}^2}} ,$$

shown in Fig. 2a (*dashed line*), where we assumed Δ_*γ*_ = −0.2%^38^. ${\epsilon _{{\rm{ff}}}}$(*A*, *γ*_e_) shows a steep slope around the ionization point, mostly caused by the *E*_*z*_-dependence of *γ*_e_ (the dependence on *A* is less significant because ${\gamma _ + }{B_0} \gg A$). Therefore, while ${E_{z}} \approx E_{z}^0$ is the fastest operation point for the flip-flop qubit driven by a resonant modulation of *A*, one might expect it to be the most prone to qubit dephasing from charge and gate noise, through the influence of *E*_*z*_ on *γ*_e_.

However, computing instead the full flip-flop qubit Hamiltonian,

7 $${{\cal H}_{{\rm{ff}}}} = {\cal H}_{{B_0}}^{{\rm{orb}}} + {\cal H}_A^{{\rm{orb}}} + {{\cal H}_{{\rm{orb}}}},$$

reveals that the qubit transition frequency has an extra bend around the ionization point (Fig. 2a, *thick yellow line*). This comes from Eq. (5), which provides a transverse coupling *g*_so_ between the flip-flop and charge qubits (*inset* in Fig. 2a):

8 $${g_{{\rm{so}}}} = \frac{A}{4}\frac{{{V_{\rm{t}}}}}{{{\epsilon _{\rm{o}}}}}$$

As a result, the electron orbit dispersively shifts the flip-flop qubit by, to second order:

9 $${D_{{\rm{orb}}}}\left( {{E_{z}}} \right) = \frac{{{{\left[ {{g_{{\rm{so}}}}\left( {{E_{z}}} \right)} \right]}^2}}}{{{\delta _{{\rm{so}}}}\left( {{E_{z}}} \right)}},$$

where *δ*_so_ = ${\epsilon _o}$ − ${\epsilon _{{\rm{ff}}}}$, reducing the flip-flop qubit frequency to:

10 $${\epsilon _{{\rm{ff}}}}\left( {A,{\gamma _{\rm{e}}},{D_{{\rm{orb}}}}} \right) = {\epsilon _{{\rm{ff}}}}\left( {A,{\gamma _{\rm{e}}}} \right) - {D_{{\rm{orb}}}}\left( {{E_{z}}} \right),$$

*D*_orb_(*E*_*z*_) is largest around ${E_{z}} \approx E_{z}^0$, since *δ*_so_ is lowest (i.e., the charge qubit frequency comes closest to the flip-flop qubit, Fig. 2a) and *g*_so_ is highest. Equation (10) (*thin black line* in Fig. 2a) agrees with full numerical simulations of the Hamiltonian in Eq. (7).

Such a dispersive shift stabilizes the flip-flop precession frequency against noise. To quantify that, we assume a quasi-static electric field noise with 100 V m^−1^ r.m.s. amplitude along the donor-dot direction (*z*-axis in Fig. 1a). This noise is equivalent to a 1.5 μeV charge detuning noise for *d* = 15 nm, consistent with experimentally observed values in similar silicon devices^39–41^—see Supplementary Note 3. The estimated—see Methods section—dephasing rates can be as low as $1{\rm{/}}T_2^ * \approx 3$ kHz (Fig. 2b), comparable to the ones due to magnetic noise ($1/T_2^ * \approx 1$ kHz in ^28^Si nanostructures^7^). This can be understood from Fig. 2c, which shows the qubit precession frequency dependence on *E*_*z*_, for three different values of *V*_t_. For small detunings *δ*_so_, i.e., *V*_t_ close to ${\epsilon _{{\rm{ff}}}}$, the dispersive shift around the ionization point is strong, yielding two first-order “clock transitions” (CT), where ∂${\epsilon _{{\rm{ff}}}}$/∂*E*_*z*_ = 0 where the dephasing rate is reduced. By increasing *V*_t_, the two first-order points merge into a single one in which both the first and second derivatives vanish, yielding the slowest qubit dephasing.

Another source of errors could come from relaxation via coupling to phonons. This is not an issue for bulk donors, where electron spin relaxation time is ${T_{1,{\rm{s}}}} \gg 1$ s^18^. However, due to the particular valley composition of the flip-flop qubit near the ionization point, its relaxation rate 1/*T*_1,ff_ due to charge-phonon coupling is enhanced^42^. We estimate it by noting that, if ${\delta _{{\rm{so}}}} \gg {g_{{\rm{so}}}}$, 1/*T*_1,ff_ is equal to the amount of charge excited state in the flip-flop eigenstates^43^ times the charge relaxation rate^42^:

11 $$1{\rm{/}}{T_{1,{\rm{ff}}}} = {\left( {{g_{{\rm{so}}}}{\rm{/}}{\delta _{{\rm{so}}}}} \right)^2}{\rm{/}}{T_{1,{\rm{o}}}},$$

12 $$1{\rm{/}}{T_{1,{\rm{o}}}} = \Theta {\epsilon _{\rm{o}}}{V_{\rm{t}}}^2,$$

where *T*_1,o_ is the charge qubit lifetime and Θ ≈ 2.37 × 10^−24^ s^2^ is determined by the silicon crystal properties^42^. Therefore, as can be seen from Fig. 2d, the higher the detuning *δ*_so_, the slower the relaxation. In particular, at the second-order CT, the qubit dephasing can be limited by relaxation, $1{\rm{/}}T_2^* = 1{\rm{/}}2{T_1} \approx {10^4}$ Hz. This limitation can be overcome by reducing *B*_0_ (Fig. 2e).

Tuning a flip-flop qubit into a clock transition requires the ability to tune the tunnel coupling *V*_t_. The latter is difficult to control at the fabrication stage, given its exponential dependence on donor depth, together with oscillations at the atomic scale^44^ arising from a similar valley interference effect as the one afflicting the exchange interaction^16^. Indeed, ion-implanting a donor at *z*_d_ ≈ 15 nm below the interface happens with a vertical uncertainty of order ±10nm^45^, resulting in more than two orders of magnitude uncertainty in *V*_t_^44^. Therefore, it is crucial to implement a method to tune *V*_t_ in situ. A possible solution is to displace the location of the interface wavefunction laterally, which in turn modifies the overlap between the donor and interface wavefunctions and therefore *V*_t_. This can be done by adding two gates on either side of the top gate, which pulls the donor electron to the interface (Fig. 2f), in such a way that a relative voltage between the gates can modify the interface lateral potential landscape. This gate stack is identical to the well-established scheme for the confinement of single electrons in Si quantum dots^10^. This technique allows *V*_t_ to be tuned by at least wo orders of magnitude (Fig. 2g), therefore circumventing the uncertainty in donor depth and *V*_t_ arising from ion-implantation.

### Adiabatic phase control

The presence of slow dephasing regions is important to control the qubit phase with high fidelity. In our quantum processor, idle qubits are decoupled from electric fields by fully displacing the electron either to the interface or to the donor. Performing quantum operations on the qubit requires displacing the electrons close to the ionization point, which in turn changes its precession frequency (Fig. 2a). As a result, the accumulated phase must be corrected after quantum operations. This is optimally done by moving the electron to the second-order clock transition, therefore minimizing dephasing errors. At this point, the flip-flop qubit phase precesses ${\rm{\&sim;}}{\Delta _\gamma }{\gamma _{\rm{e}}}{B_0}{\rm{/}}2 - {D_{{\rm{orb}}}}$ faster than its idle point, and therefore any phase correction in a 2*π* period can be applied within tens of ns. The dephasing rate at the CT, on the order of a few kHz, would cause very small errors (<10^−4^). However, while moving the electron from the interface toward the donor, the flip-flop qubit goes through regions of fast dephasing (Fig. 2b), and therefore this operation has to be performed as quickly as possible. It also has to be slow enough as to avoid erros due to non-adiabaticity, which include, e.g., leakage to unwanted high-energy states. These errors depend on the adiabatic factor *K*, which quantifies the fractional rate of change of the system’s eigenstates (the higher the value of *K*, the more adiabatic and slower is the process—see Methods section).

**Fig. 3:**
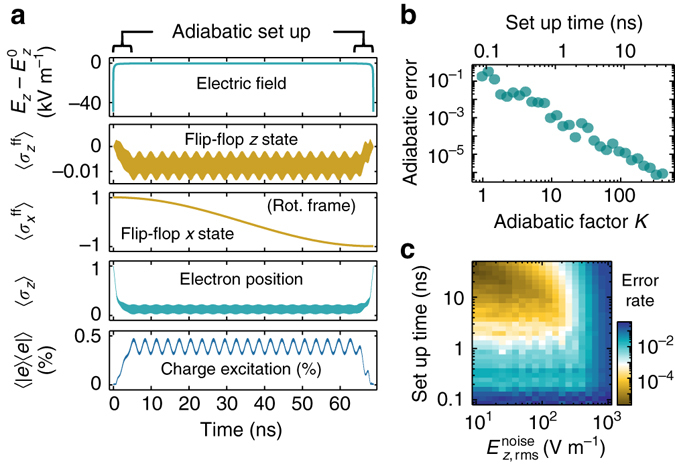
High-fidelity adiabatic *z*-gates. **a** Time-evolution of an adiabatic (*K* = 50) *π z*-gate on state $\left| g \right\rangle \otimes \left( {\left| { \downarrow \Uparrow } \right\rangle + \left| { \uparrow \Downarrow } \right\rangle } \right){\rm{/}}\sqrt 2$, showing applied electric field and flip-flop/charge states. *Outer brackets* denote the expectation value of an operator. $\sigma _{z}^{{\rm{ff}}} = \left| { \uparrow \Downarrow } \right\rangle \left\langle { \uparrow \Downarrow } \right| - \left| { \downarrow \Uparrow } \right\rangle \left\langle { \downarrow \Uparrow } \right|$ and $\sigma _x^{{\rm{ff}}} = \left| { + _x^{{\rm{ff}}}} \right\rangle \left\langle { + _x^{{\rm{ff}}}} \right| - \left| { - _x^{{\rm{ff}}}} \right\rangle \left\langle { - _x^{{\rm{ff}}}} \right|$, where $\left| { + _x^{{\rm{ff}}}} \right\rangle = \left( {\left| { \uparrow \Downarrow } \right\rangle + {\rm{exp}}\left( { - i2\pi \epsilon _{{\rm{ff}}}^{t = 0}} \right)\left| { \downarrow \Uparrow } \right\rangle } \right){\rm{/}}\sqrt 2$ and $\left| { - _x^{{\rm{ff}}}} \right\rangle = \left( {\left| { \uparrow \Downarrow } \right\rangle + {\rm{exp}}\left( { - i2\pi \epsilon _{{\rm{ff}}}^{t = 0} - i\pi } \right)\left| { \downarrow \Uparrow } \right\rangle } \right){\rm{/}}\sqrt 2$. Fast oscillations between the charge and flip-flop states are due to small deviations from perfect adiabaticity. **b** *π z*-gate leakage error for different adiabatic set-up times, which are set by the factor *K*. **c** *π z*-gate error due to quasi-static *E*_z_ noise, at the second-order CT at *B*_0_ = 0.4 T, for different noise amplitudes and adiabatic set-up times

In Fig. 3a, we plot the time dynamics of an initial state $\left| g \right\rangle \otimes \left( {\left| { \downarrow \Uparrow } \right\rangle + \left| { \uparrow \Downarrow } \right\rangle } \right){\rm{/}}\sqrt 2$ while sweeping *E*_*z*_ adiabatically (*K* = 50) to move the electron from the interface to the second-order CT and back, in order to realize a *π z*-gate. The initial adiabatic set-up part consists of a fast sweep (0.8 ns), allowed by the large charge qubit splitting when ${E_{\rm{z}}} \gg E_{\rm{z}}^0$, followed by a slower sweep (3.5 ns), limited by the proximity of excited charge states to the flip-flop qubit when ${E_{\rm{z}}} \approx E_{\rm{z}}^0$. The electron then remains at the CT for 60 ns, before adiabatically moving back to the interface. During the total 69 ns, the flip-flop qubit phase is shifted by *π*, with adiabatic errors, averaged over a set of initial flip-flop states—see Methods section—around 10^−4^. These errors can be controlled with the factor *K*, which determines the set-up time (see Fig. 3b).

Quasi-static *E*_*z*_ noise can increase errors, due to dephasing (Fig. 3c). At realistic noise levels (100 V m^−1^), the gate error rate is found to be <10^−4^. Similar error levels arise due to relaxation, which remains below 3 × 10^4^ Hz (Fig. 2d).

Note that the presence of clock transitions does not affect the ability to use *E*_ac_ to resonantly drive the qubit, since the transverse term *A*(*E*_*z*_) still responds fully to the electric field (this is similar to the case of magnetic clock transitions, e.g,. in Si:Bi^46^).

### Electric drive of the flip-flop qubit

**Fig. 4:**
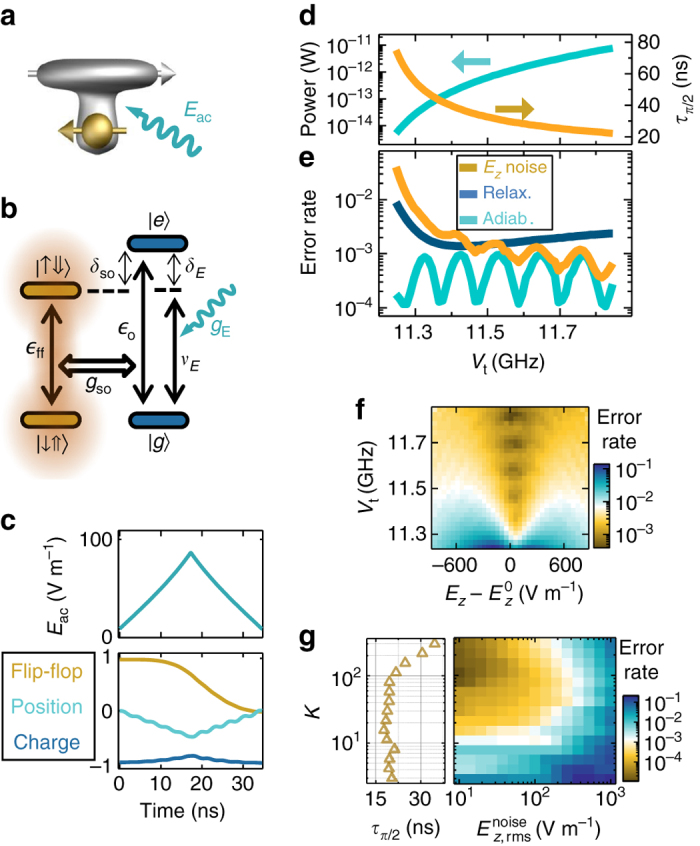
High-fidelity electrically driven adiabatic *x*(*y*)-gates. **a** Spatial representation and **b** level diagram, for electrical drive of a flip-flop qubit, showing partially ionized electron wavefunction and spin arrows. **c** Time-dependent adiabatic drive amplitude and qubit dynamics of a *π*/2 *x*-gate, for *K* = 30, *B*_0_ = 0.4 T, ${E_{\rm{z}}} = E_{\rm{z}}^0,$ and *V*_t_ = 11.5 GHz. *Bottom plot* shows flip-flop *z* state, $\left\langle {\sigma _{z}^{{ff}}} \right\rangle$, electron position, 〈*σ*_*z*_〉, and charge qubit state, $\left\langle {\left| e \right\rangle \left\langle e \right| - \left| g \right\rangle \left\langle g \right|} \right\rangle$. For the same parameters, **d** shows the averaged drive power and gate time, and **e** the error rates for different *V*_t_. To estimate the drive power, we assumed a 50 Ω line in which a 1 μV AC voltage produces a 10 V m^−1^ AC vertical electric field. **f** Estimated flip-flop qubit *π*/2 *x*-gate error due to quasi-static noise with amplitude $E_{z{\rm{,rms}}}^{{\rm{noise}}} = 100$ V m^−1^. **g** Dependence of gate error rate on the electric noise r.m.s. amplitude and adiabatic factor *K* (which sets the gate time)

We now explain how high-fidelity one-qubit *x*(*y*)-gates can be achieved via electric drive of the flip-flop qubit. The fastest one-qubit gates are obtained when the electron is around the ionization point, where ∂*A*/∂*E*_z_ is maximum (Fig. 1d). A vertical oscillating electric field of amplitude *E*_ac_ is applied (Fig. 4a) in resonance with the flip-flop qubit, i.e., *ν*_E_ = ${\epsilon _{{\rm{ff}}}}$. A large detuning ${\delta _{{\rm{so}}}} \gg {g_{{\rm{so}}}}$ (Fig. 4b) ensures the least amount of the charge excited state $\left| e \right\rangle$ in the qubit eigenstates, minimizing qubit relaxation via charge-phonon coupling. The flip-flop qubit is still driven, via a second-order process, at a rate (half-Rabi frequency):

13 $$g_{\rm{E}}^{{\rm{ff}}} = \frac{{{g_{{\rm{so}}}}{g_{\rm{E}}}}}{2}\left( {\frac{1}{{{\delta _{{\rm{so}}}}}} + \frac{1}{{{\delta _{\rm{E}}}}}} \right),$$

where *δ*_E_ = *ν*_E_ − ${\epsilon _o}$ and *g*_E_ is the driven electric coupling rate between the two charge eigenstates:

14 $${g_{\rm{E}}} = \frac{{e{E_{{\rm{ac}}}}d}}{{4h}}\frac{{{V_{\rm{t}}}}}{{{\epsilon _{\rm{o}}}}},$$

where *E*_ac_ is the amplitude of a sinusoidal drive. Equation (13) provides another explanation of why the fastest one-qubit gates are obtained when the electron is at the ionization point: *δ*_so_ and *δ*_E_ are minimum (${\epsilon _o}$ is minimum), and *g*_so_ and *g*_E_ are maximum (Eqs. (8) and (14)).

The electrical drive can cause some excitation of the charge qubit. It is therefore convenient to turn *E*_ac_ on/off adiabatically to make sure the charge is de-excited at the end of the gate. Figure 4c shows the *E*_ac_ time evolution needed for a *π*/2 *x*-gate, where we have assumed an adiabatic factor *K* = 30, sufficient for leakage errors <10^−3^. *E*_ac_ increases steadily until a *π*/4 rotation is completed, after which *E*_ac_ is gradually switched off to achieve an adiabatic *π*/2 *x*-gate. An average 4% excitation of the charge qubit causes a ~4 × 10^4^ Hz relaxation rate of the encoded quantum state (Eq. 12), or error levels close to 10^−3^.

We then investigate how the total *π*/2 *x*-gate errors depend on the biasing of the electron wavefunction. At the ionization point, $E_{z} = E_{z}^0$, error levels close to 10^−3^ are found over a wide range of *V*_t_ (Fig. 4e). The *K* = 30 choice ensures adiabatic errors <10^−3^ with an oscillatory character typical of adiabatic processes^47^. At small *V*_t_ (and therefore small detuning *δ*_so_), the qubit eigenstates contain a substantial amount of charge, causing more errors due to charge-phonon relaxation. Increasing the detuning *δ*_E_ with larger *V*_t_ allows for a faster adiabatic sweep and higher powers (Fig. 4d), yielding shorter gate times and therefore less errors due to quasi-static noise. Still, the incident power is at least three orders of magnitude lower than the one needed to drive donor electron spin qubits, at the same Rabi frequency, with oscillating magnetic fields^7, 19^.

As Fig. 4f shows, low error rates are still available away from the ionization point, even though best values are found at ${E_{z}} = E_{z}^0$. This is because our gate times are so fast that dephasing, and therefore CTs, do not play a crucial role. Instead, quasi-static *E*_z_ noise cause errors mainly by modulating the driving strength $g_{\rm{E}}^{{\rm{ff}}}$, causing “gate time jitter”. Indeed, the gate time is sensitive to the orbital transition frequency ${\epsilon _o}$ (Eq. 13), and therefore gate errors are minimized close to the charge qubit sweet spot (CQSS), where ∂${\epsilon _o}$/∂*E*_*z*_ = 0 (Fig. 2a).

Finally, as Fig. 4g shows, lower quasi-static *E*_z_ noise can cause less errors, provided that the adiabatic factor *K* is increased, to reduce leakage errors, up to an optimum value where gate times are still fast as to keep noise errors low. Relaxation errors could also be reduced by reducing *B*_0_ (recall Fig. 2e).

A number of other noise sources, including high frequency charge noise, Johnson-Nyquist, and evanescent-wave Johnson noise^48^ (EWJN) also affect qubits that are sensitive to electric fields. However, as we discuss in Supplementary Note 3, the corresponding error rates are much lower than the ones already previously mentioned—see all estimated error levels in Table 1.

**Table 1:** Error rates of ***x***(***y***)-gates from different noise sources as discussed in Supplementary Note 3

**Noise source**	**Spectral bandwidths**		
	**Quasi static (<10** ^**6**^ ** Hz)**	**Rabi (~10** ^**7**^ ** Hz)**	**Qubit (~10** ^**10**^ ** Hz)**
1/*f* vertical (*E*_*z*_)	10^−3^	<10^−4^	10^−4^
1/*f* horizontal (*E*_*x,y*_)	10^−4^	<10^−5^	-
Charge-phonon relaxation	-	-	10^−3^
Johnson-Nyquist	≪10^−5^	<10^−5^	<10^−4^
EWJN	-	<10^−6^	<10^−4^

Hyphens indicate non-existent or negligible errors.

### Two-qubit coupling via electric dipole interaction

**Fig. 5:**
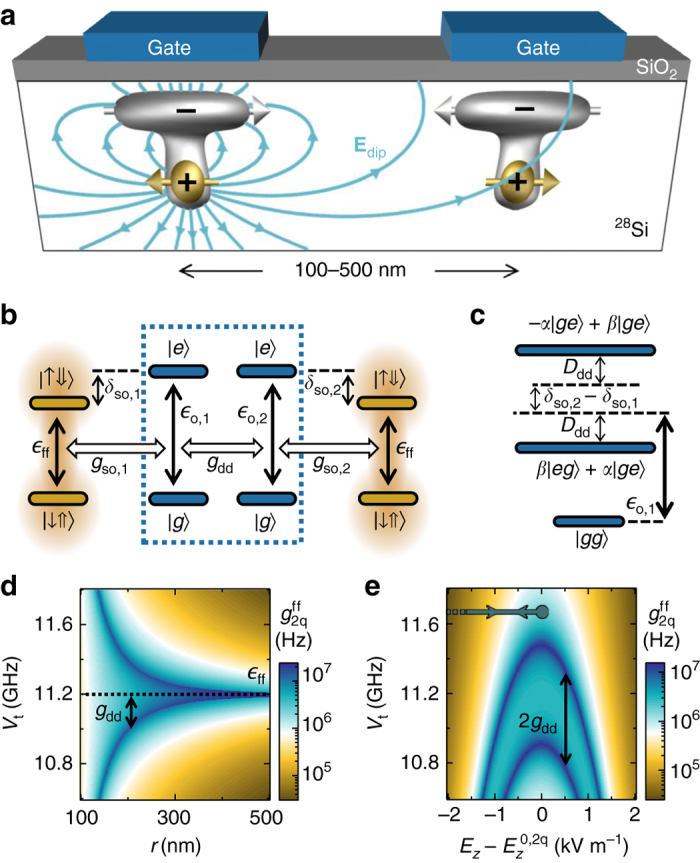
Robust electric dipole-dipole interactions between two distant flip-flop qubits. **a** Device scheme for coupling qubits, showing dipole field lines, **E**_dip_, produced by the dipole on the left. **b** Level diagram for two-qubit coupling via electric dipole-dipole interaction. **c** Lowest molecular eigenstates for the two charge qubits inside *dashed rectangle* in **b**. The eigenenergy shift equals *D*_dd_ = (*δ*_so,2_ − *δ*_so,1_)$(\sqrt {1+[2g_{dd}/({\delta _{{\rm{so}},2}}-{\delta _{{\rm{so}},1}})]^2}-1)/2$. The eigenstate coefficients are $\beta = \theta {\rm{/}}\sqrt {{\theta ^2} + 1}$ and $\alpha = \phi {\rm{/}}\sqrt {{\phi ^{\rm{2}}}{\rm{ + 1}}}$, with $\theta ,\phi = \left[ {\left( {{\delta _{{\rm{so}},2}} - {\delta _{{\rm{so}},1}}} \right) \pm \sqrt {{{\left( {{\delta _{{\rm{so}},2}} - {\delta _{{\rm{so}},1}}} \right)}^2} + {{\left( {2{g_{{\rm{dd}}}}} \right)}^2}} } \right]{\rm{/}}\left( {2{g_{{\rm{dd}}}}} \right)$. Effective coupling between two flip-flop qubits as a function of *V*_t,1_ = *V*_t,2_ = *V*_t_, interdistance *r* (**d**) and electric field *E*_*z*,1_ = *E*_*z*,2_ = *E*_z_ (**e**). The *arrows* in **e** represent the adiabatic path followed for two-qubit gates. $E_{z}^{{\rm{0}},2{\rm{q}}}$ is the ionization point in the presence of a second qubit, $E_{z}^{{\rm{0,2q}}} = E_{z}^0 - 2{g_{{\rm{dd}}}}h{\rm{/}}\left( {2e{d_{\rm{i}}}} \right)$

We now present the method to couple donor spins that lies at the heart of our scalable quantum processor. It exploits the electric dipole that naturally arises when a donor-electron wavefunction is biased to the ionization point (Fig. 5a), due to the fact that a negative charge has been partly displaced away from the positive ^31^P nucleus. The electric field produced by this induced dipole in turn, modifies the energy of a nearby donor which is also biased at the ionization point, resulting in a long-range coupling between the two.

The interaction energy between two distant dipoles, *μ*_1_ and *μ*_2_, oriented perpendicularly to their separation, *r*, is^49^ ${V_{{\rm{dip}}}} = {\mu _1}{\mu _2}{\rm{/}}\left( {4\pi {\varepsilon _{\rm{r}}}{\varepsilon _0}{r^3}} \right)$, where *ε*_0_ is the vacuum permittivity and $\epsilon$_r_ the material’s dielectric constant (*ε*_r_ = 11.7 in silicon). The electric dipole of each donor-interface state is *μ*_*i*_ = *ed*_i_(1 + *σ*_*z*,i_)/2, implying that the dipole-dipole interaction Hamiltonian is:

15 $${{\cal H}_{{\rm{dip}}}} = {V_{{\rm{dd}}}}\left( {{\sigma _{{\rm{z}},1}}{\sigma _{{\rm{z}},2}} + {\sigma _{{\rm{z}},1}} + {\sigma _{{\rm{z}},2}}} \right)$$

16 $${V_{{\rm{dd}}}} = \frac{1}{{16\pi {\varepsilon _0}{\varepsilon _{\rm{r}}}h}}\frac{{{e^2}{d_1}{d_2}}}{{{r^3}}}$$

This electric dipole-dipole interaction is therefore equivalent to a small shift in the equilibrium orbital position of both electrons plus a coupling term between the charge qubits (*blue dashed rectangle* in Fig. 5b) equal to:

17 $${g_{{\rm{dd}}}} = {V_{{\rm{dd}}}}\frac{{{V_{{\rm{t}},1}}{V_{{\rm{t}},2}}}}{{{\epsilon _{{\rm{o}},1}}{\epsilon _{{\rm{o}},2}}}}$$

Note that this interaction can be stronger due to the presence of a metallic interface on top of the qubits, which enhances vertical dipoles—see Supplementary Note 2. Most importantly, since each flip-flop qubit is coupled to their electron position (Eq. 5), the electric dipole-dipole interaction provides a natural way to couple two distant flip-flop qubits.

Indeed, the effective coupling rate between two flip-flop qubits at the ionization point, Fig. 5d, exceeds 10 MHz around two narrow regions. These bands can be understood from the energy-level diagram shown in Fig. 5c. The two charge qubits in Fig. 5b form hybridized molecular states, which are coupled to each flip-flop qubit. The two-qubit coupling rate is maximum when in resonance with a molecular state. However, this regime induces too many relaxation errors due to resonant charge excitation. Therefore, it is best to detune the flip-flop qubits from the molecular states, while still keeping a substantial inter-qubit coupling rate, via a second-order process, equal to:

18 $$g_{{\rm{2q}}}^{{\rm{ff}}} = {g_{{\rm{so,1}}}}{g_{{\rm{so,2}}}}\alpha \beta \left( {\frac{1}{{{D_{{\rm{dd}}}} - {\delta _{{\rm{so}},1}}}} + \frac{1}{{{D_{{\rm{dd}}}} + {\delta _{{\rm{so,2}}}}}}} \right),$$

where *D*_dd_ is the charge eigenenergies shift and *α*, *β* the eigenstates coefficients—see Fig. 5c caption.

**Fig. 6:**
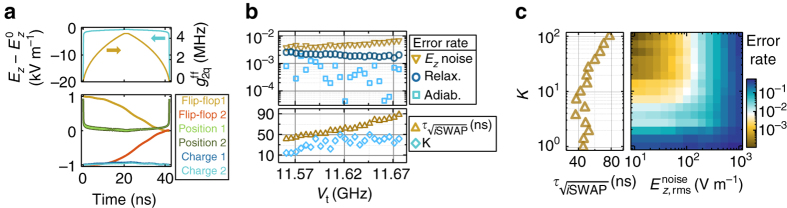
High-fidelity adiabatic $\sqrt {i{\rm{SWAP}}}$ gates between two distant flip-flop qubits. **a** Time evolution of an adiabatic $\sqrt {i{\rm{SWAP}}}$ gate, for *K* = 30, *r* = 180 nm, *B*_0_ = 0.4 T, and *V*_t_ = 11.58 GHz. **b** Optimized $\sqrt {i{\rm{SWAP}}}$ gate error, gate time, and adiabatic factor *K*. **c** Optimized error rate arising from quasi-static *E*_*z*_-noise, for different noise amplitudes and adiabatic factor *K* (which sets the gate time)

Two-qubit gates start with both electrons at the interface, where qubits are decoupled since the electric dipoles and the hyperfine interactions are first-order insensitive to vertical electric fields. Indeed, from Eq. (18), $g_{{\rm{2q}}}^{{\rm{ff}}}$ is negligible since *g*_so_ vanishes and *δ*_so_ diverges. The electrons are then simultaneously and adiabatically displaced to the ionization point for a time necessary for an $\sqrt {i{\rm{SWAP}}}$ gate, before returning to the interface. In Fig. 6a, we show the dynamics of a two-qubit gate performed with an adiabatic factor *K* = 30, following the trajectory shown in Fig. 5e. Similarly to one-qubit *z*-gates, the electron is first displaced in a fast time scale (~0.3 ns) set by the charge qubit parameters (${\epsilon _0}$ and *V*_t_), followed by a slower sweep (~19 ns) set by the spin-charge coupling parameters (*δ*_so_ and *g*_so_), until it reaches the ionization point. The electron remains still for a short time before the whole process is then reversed. In the end, a $\sqrt {i{\rm{SWAP}}}$ gate is performed. While some amount of charge is excited during the process, it goes back to its ground state, $\left| {gg} \right\rangle$, with an adiabatic error around 10^−3^.

We quantify the two-qubit gate fidelity in presence of the most deleterious noise types for our qubits, namely quasi-static *E*_z_ noise and charge-phonon relaxation. For this, we observe that the optimal gate fidelities are achieved when ${E_{z}}\left( {{\tau _{\sqrt {i{\rm{SWAP}}} }}{\rm{/}}2} \right) \approx E_{z}^0$. Similarly to one-qubit *x*-gates, this happens because $\sqrt {i{\rm{SWAP}}}$ gates are sensitive to gate time jitter, and therefore errors are minimized at the CQSS, where $g_{{\rm{2q}}}^{{\rm{ff}}}$ is robust against *E*_z_ noise to first order—recall Fig. 5e and Eq. (18). An optimization algorithm finds the best adiabatic factor *K* that minimizes errors due to *E*_z_ noise for each value of *V*_t,1_ = *V*_t,2_ = *V*_t_. The result is shown in Fig. 6b. Smaller detunings *δ*_so_ (small *V*_t_) result in shorter gate times, which in turn reduces errors from quasi-static noise. However, this also implies a larger admixture of charge in the qubit eigenstates, which slightly increases relaxation errors. The lowest error rates, ~3 × 10^−3^ are found at small detunings, *V*_t_ − ${\epsilon _{{\rm{ff}}}}$ − *g*_dd_ ≈ 100 MHz (*V*_t_ ≈ 11.59 GHz). At even smaller detunings, the two-qubit coupling rate becomes too fast, requiring faster adiabatic sweeps to avoid over-rotation (lower *K*, Fig. 6b) and generating more leakage errors. The gate errors remain within 10^−3^ − 10^−2^ for a wide range of *V*_t_. Finally, we estimate in Fig. 6c how noise errors depend on the noise amplitude and adiabatic factor *K*, which sets the gate time.

Our proposed two-qubit gates are not only well protected against noise, but also robust against donor misplacement. Variations in *r*, *d*_1_, and *d*_2_ mainly cause variations in the charge qubits coupling *g*_dd_, therefore simply changing the energy separation between molecular charge states (Fig. 5c). However, the coupling $g_{{\rm{2q}}}^{{\rm{ff}}}$ between the flip-flop qubits can be kept essentially constant by simply readjusting *V*_t_, using, e.g., the method described in Fig. 2f, g. Figure 5d shows that one can keep a constant value of, e.g., $g_{{\rm{2q}}}^{{\rm{ff}}} = 1$ MHz for any inter-donor spacing between 180 and 500 nm, by adjusting *V*_t_ between 11.3 and 11.8 GHz. In other words, since the flip-flop qubit coupling is mediated by a tunable interaction with their respective charge qubits, the inter-qubit interaction does not need to decay with *r* ^3^, as one would otherwise get when the dipole interaction couples the qubits directly^26, 31^. Therefore, two-qubit operations can be turned on between pairs of qubits separated by many sites in a two-dimensional array. This tunable long-range connectivity can be exploited to great advantage in large-scale quantum processors^50^. The large tolerance in *g*_dd_ also accommodates very well the donor depth uncertainties inherent to ion implantation^45^, given the linear dependence of $g_{{\rm{2q}}}^{{\rm{ff}}}$ on *d*_i_ (Eqs. (16) and (17)).

We conclude that our scheme provides a dramatic reduction in the fabrication complexity, especially compared to schemes that require placing a gate between a pair of tightly spaced donors, such as the Kane’s proposal^15^, which requires *r* ≈ 15 nm separation between two ^31^P nuclear spins. Note that, by relocating the problem of valley oscillations from the exchange interaction^15^ to the tunnel coupling, we have effectively provided a way in which the delicate parameter can now be tuned using a much simpler gate geometry.

### Scaling up using circuit quantum electrodynamics

**Fig. 7:**
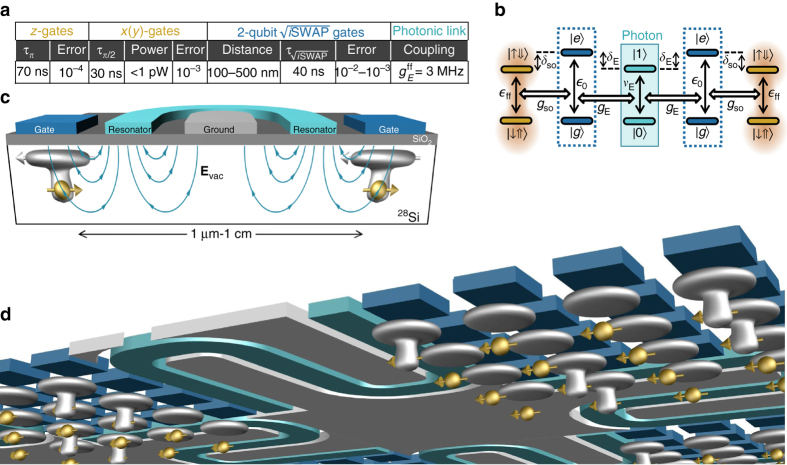
Silicon hybrid quantum processor. **a** Figures of merit summarizing the speed and error rates of different gate schemes presented in this paper, assuming realistic noise sources. **b** Level diagram for distant flip-flop qubit coupling via a microwave resonator showing photon number states and off-resonant charge states. **c** Device scheme for coupling qubits via a photonic link. Distant donors, placed next to the resonator center line and biased to their ionization point, are subject to the vacuum electric field **E**_vac_ of a shared microwave resonator. **d** Schematic view of a large-scale quantum processor based upon ^31^P donors in Si, operated and coupled through the use of an induced electric dipole. Idle qubits have electrons at the interface, leaving the ^31^P nucleus in the ultra-coherent ionized state. Electrons are partially shifted toward the donor for quantum operations. The *sketch* shows a possible architecture where a cluster of qubits is locally coupled via the electric dipole, and a subgroup thereof is further coupled to another cluster through interaction with a shared microwave cavity (*aqua*). The drawing is not to scale; control lines and readout devices are not shown

In order to reach the long-term goal of a large-scale quantum processor, wiring up the control and read-out lines for each individual qubit is not trivial, given the high density in typical spin qubit architectures^51^. Recent solutions include cross-wiring using multilayer lithography^26^ or floating gate electrodes inspired by dynamic random access memory systems^52^. In both cases, using flip-flop qubits with long-distance interactions would result in widely spaced donors and loose fabrication tolerances. In addition, since flip-flop qubits are coupled via electric fields, they could be spaced further apart by using electrical mediators. These include floating metal gates^53^ or even microwave resonators. Indeed, the use of electric dipole transitions allows a natural integration of donor-based spin qubits into a circuit-quantum electrodynamics architecture^43, 54–56^ (see Fig. 7c for a possible device layout).

A full quantum mechanical treatment yields a charge-photon coupling rate given by Eq. (14), with *ν*_E_ now representing the resonator fundamental mode frequency and *E*_ac_ the resonator vacuum field, **E**_vac_. Again, it is best to have the charge-excited state detuned from the flip-flop transition and resonator photon (see Fig. 7b), therefore minimizing charge excitation while retaining a second-order flip-flop photon coupling given by Eq. (13). Assuming *δ*_so_ ≈ *δ*_E_ ≈ 10*g*_so_ ≈ 10*g*_E_, a *d* = 15 nm deep ^31^P flip-flop qubit would be coupled to photons at a $g_{\rm{E}}^{{\rm{ff}}} \approx 3$ MHz rate. This is three orders of magnitude faster than the electron-spin coupling rate to a resonator via its magnetic vacuum field^57, 58^, and comparable to the coupling strength obtained by using strong magnetic field gradients^59, 60^, but without the need to integrate magnetic materials within a superconducting circuit. This assumes a vacuum field amplitude **E**_vac_ ≈ 30 V m^−1^, which can be obtained by using tapered coplanar waveguide or high-inductance resonators^61^.

The possibility of coupling the qubits to microwave photons provides a path for dispersive qubit readout, as well as for photonic interconnects. Near-quantum limited amplifiers have recently become available to obtain excellent read-out speed and fidelities^62^. The resonator can also be used as a quantum bus to couple two spin qubits separated by as far as 1 cm (Fig. 7c), a distance given by the mode wavelength. Figure 7b shows the detailed energy-level diagram. To avoid losses from photon decay, the qubits should be detuned from the resonator by an amount much greater than the qubit-photon coupling rates. Assuming $\delta _{\rm{E}}^{{\rm{ff}}} = 10g_{\rm{E}}^{{\rm{ff}}}$, where $\delta _{\rm{E}}^{{\rm{ff}}} = {\nu _{\rm{E}}} - {\epsilon _{{\rm{ff}}}}$, the effective two-qubit coupling $g_{{\rm{2q}}}^{{\rm{ff}}} \approx {\left( {g_{\rm{E}}^{{\rm{ff}}}} \right)^2}{\rm{/}}\delta _{\rm{E}}^{{\rm{ff}}} \approx 0.3$ MHz yields a $\sqrt {i{\rm{SWAP}}}$ gate that takes only 0.4 μs.

## Discussion

Figure 7a summarizes the key figures of merit of a quantum processor based on flip-flop qubits coupled by electric dipole interactions. Fast one-qubit *x*(*y*)-gates are attainable with low electric drive power and error rates ~10^−3^. Two-qubit $\sqrt {i{\rm{SWAP}}}$ gates are fast and with error rates approaching 10^−3^. At the end of all operations, the phase of each qubit can be corrected, via adiabatic *z*-gates, in fast time scales and low error rates ~10^−4^. These values are based on current experimentally known values of charge noise in silicon devices^39^, and are possibly amenable to improvement through better control of the fabrication parameters. More advanced control pulse schemes could allow for faster gates with less leakage^63–65^, and active noise cancellation techniques, e.g., pulses for gate time jitter^66^ or decoherence^67^ suppression, could further improve gate fidelities.

Idle qubits are best decoupled from all other qubits by having the electron at the interface and the quantum state stored in the nuclear spin, which has a record coherence times *T*_2_ ≳ 30 s^7^, and can be even longer in bulk samples^6^. Quantum information can be swapped between the nuclear and the flip-flop qubit by simply applying an ESR *π*-pulse that excites the $\left| { \downarrow \Downarrow } \right\rangle$ state to $\left| { \uparrow \Downarrow } \right\rangle$ (Fig. 1c).

Qubit read-out can be obtained by spin-dependent tunneling into a cold charge reservoir, detected by a single-electron transistor^18^. Read-out times can be ~1 μs with cryogenic amplifiers^68^, which is comparable to the time necessary to perform, e.g., ~20 individual gates lasting ~50 ns each, in a surface code error correction protocol^2^.

A large-scale, fault-tolerant architecture can be built in a variety of ways. One- or two-dimensional arrays can be built to implement error correction schemes such as the Steane^69^ or the surface^2^ code, since all mutual qubit couplings are tunable and gateable. A larger processor can include a hybrid of both coupling methods, incorporating cells of dipolarly coupled qubits, interconnected by microwave photonic links (Fig. 7d), in which case more advanced error-correction codes can be implemented^1, 3, 4, 50^. Microwave resonators could be also used to interface donors with superconducting qubits^8, 70^, for the long-term goal of a hybrid quantum processor that benefits from the many advantages of each individual architecture^55^.

In conclusion, we have presented a way to encode quantum information in the electron-nuclear spin states of ^31^P donors in silicon, and to realize fast, high-fidelity, electrically driven universal quantum gates. Our proposal provides a credible pathway to the construction of a large-scale quantum processor, where atomic-size spin qubits are integrated with silicon nanoelectronic devices, in a platform that does not require atomic-scale precision in the qubit placement. The qubits are naturally amenable to being placed on two-dimensional grids and, with realistic assumptions on noise and imperfections, are predicted to achieve error rates compatible with fault-tolerant quantum error correction.

## Methods

### Adiabaticity

Given a time-dependent Hamiltonian in a two-dimensional Hilbert space,

19 $${{\cal H}_2} = \Delta (t){\sigma _{z}} + \Omega (t){\sigma _{x}},$$

in units of rad s^−1^, the adiabatic condition is expressed as^71^

20 $$K = \left| {\frac{{{\omega _{{\rm{eff}}}}}}{{\dot \alpha }}} \right| \gg 1,$$

where ${\omega _{{\rm{eff}}}} = \sqrt {{\Delta ^2} + {\Omega ^2}}$ is the instantaneous transition angular frequency between eigenstates, and $\dot \alpha$ is the rate of change of the orientation of *ω*_eff_ (*α* = arctan(Ω/Δ)). It follows from Eq. (20) that

21 $$K = \frac{{{{\left( {{\Delta ^2} + {\Omega ^2}} \right)}^{3/2}}}}{{\left| {\dot \Delta \Omega - \dot \Omega \Delta } \right|}} \gg 1,$$

Although the processes described in this paper involve multiple levels, we applied Eq. (21) in different forms as an approximation of adiabaticity. This was confirmed to be always valid by checking that the leakage errors were kept below a target level.

In particular, for one-qubit *z*-gates and two-qubit $\sqrt {i{\rm{SWAP}}}$ gates, we used ${\Delta _{\rm{c}}} = \pi e\left( {{E_{z}} - E_{z}^0} \right)d{\rm{/}}h$ and Ω_c_ = *πV*_t_ to find *K*_c_ for the charge qubit, and Δ_so_ = *πδ*_so_ and Ω_so_ = 2*πg*_so_ to find *K*_so_ for the spin-charge coupling. For a chosen adiabatic factor *K*, we find *E*_z_(*t*) by satisfying the condition min(*K*_so_, *K*_c_) = *K*.

For one-qubit drive, we used Δ_E_ = *πδ*_E_ and Ω_E_ = 2*πg*_E_ to find *K*_E_. A particular choice of *K* = *K*_E_ sets the adiabatic sweep rate of *E*_ac_(*t*).

### Estimation of dephasing and gate errors

In order to estimate the effects of quasi-static *E*_z_ noise on dephasing, we first calculate the flip-flop qubit transition frequency ${\epsilon _{{\rm{ff}}}}$ (difference between eigenfrequencies corresponding to eigenstates closest to $\left| {g \downarrow \Uparrow } \right\rangle$ and $\left| {g \uparrow \Downarrow } \right\rangle$, which we denote as ${\left| {g \downarrow \Uparrow } \right\rangle _{\rm{e}}}$ and ${\left| {g \uparrow \Downarrow } \right\rangle _{\rm{e}}}$). Next, for a uniformly distributed noise in the range $E_{z}^n = \sqrt 3 \left[ { - E_{z,{\rm{rms}}}^{{\rm{noise}}},E_{z,{\rm{rms}}}^{{\rm{noise}}}} \right]$, we estimate the qubit dephasing rate to be

22 $${\rm{Dephasing}}\,{\rm{rate}} = \mathop {\sum}\limits_n {\left| {{\epsilon _{{\rm{ff}}}} - \epsilon _{{\rm{ff}}}^n} \right|{\rm{/}}{N_n}} ,$$

where *N*_*n*_ is the number of sampled $E_{z}^n$ and $\epsilon _{{\rm{ff}}}^n$ is calculated for each value of $E_{z}^n$.

The averaged error rate (without noise) of a desired adiabatic unitary process *U*_ideal_ is calculated by averaging the fidelity of the actual process *U* over a set of initial states $\left| j \right\rangle$,

23 $${\rm{Adiabatic}}\,{\rm{error}} = 1 - \mathop {\sum}\limits_{\left| j \right\rangle } {{{\left| {\left\langle j \right|{U^\dag }{U_{{\rm{ideal}}}}\left| j \right\rangle } \right|}^2}{\rm{/}}{N_j}} ,$$

where *N*_*j*_ is the number of initial states. For one-qubit gates (e.g., a *π* z-gate or a *π*/2 *x*(*y*)-gate), we choose $\left| j \right\rangle = \left\{ {{{\left| {g \downarrow \Uparrow } \right\rangle }_{\rm{e}}},{{\left| {g \uparrow \Downarrow } \right\rangle }_{\rm{e}}},\left( {{{\left| {g \downarrow \Uparrow } \right\rangle }_{\rm{e}}} + {{\left| {g \uparrow \Downarrow } \right\rangle }_{\rm{e}}}} \right){\rm{/}}\sqrt 2 ,\left( {{{\left| {g \downarrow \Uparrow } \right\rangle }_{\rm{e}}} + i{{\left| {g \uparrow \Downarrow } \right\rangle }_{\rm{e}}}} \right){\rm{/}}\sqrt 2 } \right\}$ and *N*_*j*_ = 4, whereas for two-qubit gates (e.g., $\sqrt {i{\rm{SWAP}}}$) $\left| j \right\rangle = \left| {{j_1}} \right\rangle \otimes \left| {{j_2}} \right\rangle$ (the 1,2 indexes refer to the aforementioned four initial states for each qubit) and *N*_*j*_ = 16.

To estimate the averaged gate error rate under quasi-static *E*_z_ noise, the actual process *U* and eigenstates $\left| j \right\rangle$ are calculated for each value of $E_{\rm{z}}^n$ before averaging,

24 $${\rm{Noise}}\,{\rm{error}} = 1 - \mathop {\sum}\limits_{n,{{\left| j \right\rangle }_n}} {{{\left| {{{\left\langle j \right|}_n}U_n^\dag {U_{n,{\rm{ideal}}}}{{\,\left| j \right\rangle }_n}} \right|}^2}{\rm{/}}\left( {{N_j}{N_n}} \right)} $$

Finally, to estimate errors due to charge-phonon relaxation, we multiply the averaged charge excitation by its relaxation rate and assume a exponential decay in fidelity:

25 $${\rm{Relax}}{\rm{.}}\,{\rm{error}} = \frac{{1 - {\rm{e}^{ - \mathop {\int}\limits_0^{{\tau _{{\rm{gate}}}}} {\left( {\mathop {\sum}\limits_{\left| {j(t)} \right\rangle } \left\langle {j(t)\left| e \right\rangle \left\langle e \right|j(t)} \right\rangle {\rm{/}}{N_j}} \right){\rm{d}}t/{T_{1,{\rm{o}}}}} }}}}{2},$$

where $\left| j (t)\right\rangle$ are the time-evolution of the initial set states $\left| j \right\rangle$. For two-qubit gates, we sum up the error rate of each qubit.

### Data availability

The data that support the findings of this study are available from the corresponding author on reasonable request.

## Electronic supplementary material

Supplementary Information
