# Supplementary material for: Silicon quantum processor with robust long-distance qubit couplings
Source: Nat Commun. 2017 Sep 6;8:450. doi: 10.1038/s41467-017-00378-x (PMC5587611; doi:10.1038/s41467-017-00378-x)
Supplement: Supplementary file 1 — Supplementary Information [file 41467_2017_378_MOESM1_ESM.pdf]

Description of Supplementary Files

File Name: Supplementary Information
Description: Supplementary Figures, Supplementary Notes and Supplementary References

Supplementary Note 1. Validity of the two-level approximation for the electron orbital wavefunction

The concepts and calculations shown in the manuscript are based upon approximating the electron orbital degree of freedom as a two-level system, i.e. a charge qubit. The true orbital levels of a donor-interface system are, of course, more complex than that. However, below we show that the charge qubit model represents an excellent approximation, for the range of parameters relevant to our proposal.

The ground orbital wavefunction $|d\rangle$ of an electron bound to a donor is a symmetric combination of the 6 conduction band minima ("valleys") ($k_{\pm x}$, $k_{\pm y}$, $k_{\pm z}$) in silicon[1]. Higher excited valley-orbit states are separated by > 10 meV and can be safely neglected. Conversely, the orbital states of an electron confined at the Si/SiO_2 interface comprise a low-energy doublet of states, with wavefunctions constructed as a combination of the $k_{\pm z}$ valleys. The k_{+z} and k_{-z} valleys are coupled by the abrupt potential of the interface, which breaks the degeneracy of the ground state doublet into the lower valley $|i\rangle$ and upper valley $|v\rangle$ states, separated by the valley splitting V_s (ref. 2). All the remaining excited donor and interface orbital states are well above the ground doublet by several meV[3,4]. When the donor is close to ionization, the lowest-energy states of the system therefore consist of $|d\rangle$, $|i\rangle$ and $|v\rangle$ states, as shown in Supp. Fig. 1 inset.

We computed the above three energy levels with the atomistic tight binding package NEMO-3D[5,6], assuming a donor placed at depth $z_d = 15.2$ nm below the Si/SiO_2 interface, and biased close to the donor ionization field E_z^0. The dependence of the energies of $|d\rangle$, $|i\rangle$ and $|v\rangle$ on electric field E_z is shown by the dots in Supp. Fig. 1. We also fit the lowest energy levels with the charge qubit two-level model described by the Hamiltonian $\mathcal{H}_{\rm orb}$ (in Eq. 2 of the main manuscript), and plot them as solid blue lines in Supplementary Fig. 1. The two-level model agrees well with tight-binding calculation taking $V_t = 9.3$ GHz and $d = 11$ nm in Eq. 2. Here, d represents the separation between the center-of-mass positions of the donor-bound ($|d\rangle$) and interface-bound ($|i\rangle$) orbitals. This is the relevant quantity in calculating the electric dipole strength. The extracted value d is lower than the donor depth z_d, as expected, and is consistent with the separation between the mean positions of the donor and interface electron wavefunctions as modeled with NEMO-3D.

Supplementary Fig. 1 shows that, when $E_z \ll E_z^0$, the orbital ground state $|g\rangle$ of the electron is localized at the donor, whereas the first excited state corresponds to the lower valley interface state. The two states are separated in energy by ϵ_o, given by Eq. 3 of the main manuscript. As E_z increases, the two states approach, and anticross at $E_z = E_z^0$. For $E_z \gg E_z^0$, the donor state will eventually (at $E_z^v \sim 4.11$ MV m^{-1}) anticross with the upper valley interface state. Therefore, as shown by the solid lines in Supp. Fig. 1, a two-level model described by the

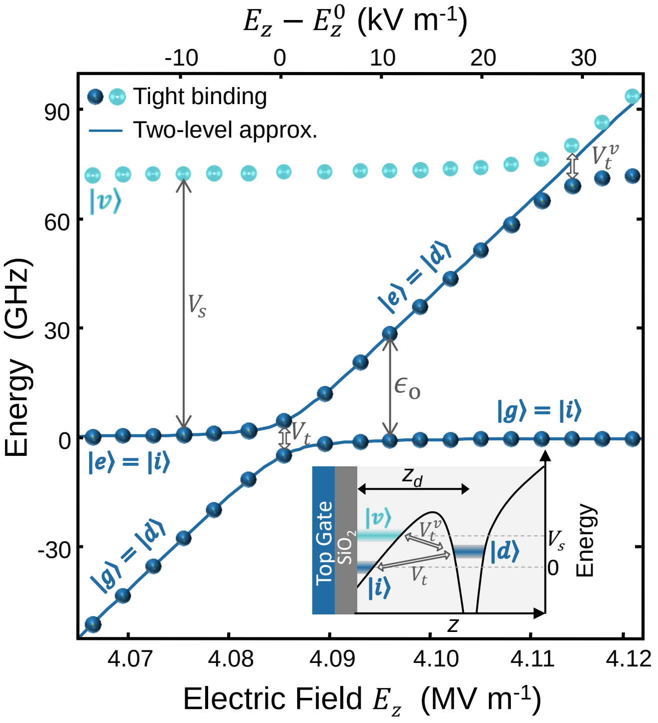

Supplementary Figure 1. **Orbital and valley states.** The lowest orbital energy levels of the donor-interface system, with respect to the lower valley interface state $|i\rangle$ (set as the zero-energy reference). The donor is assumed 15.2 nm below a Si/SiO_2 interface. The dots correspond to the energy levels obtained from a full-scale tight-binding calculation with NEMO-3D. Solid lines represent the energy levels obtained from the two level approximation described by Eq. 2 in the main manuscript. An excellent agreement between our two-level model and tight binding calculations is observed, since the valley splitting V_s is much larger than the tunnel coupling V_t. Inset: Potential profile as a function of depth, illustrating the donor $|d\rangle$, lower $|i\rangle$ and upper $|v\rangle$ valley interface states. The donor ground state is tunnel-coupled to the lower and upper valley interface states by V_t and V_t^v respectively.

$|d\rangle$ and $|i\rangle$ states constitutes an excellent approximation for $E_z < E_z^v$. This allows a broad range of validity of the simple charge qubit model, provided the interface valley splitting V_s is much larger than the tunnel coupling V_t. The NEMO-3D model used here predicts $V_s = 71.7$ GHz, which is indeed much larger than $V_t = 9.3$ GHz. Experimentally, even higher values of V_s are routinely observed in electrons confined at the Si/SiO_2 interface by top-gated structures[7], providing further reassurance on the practical validity of our models.

Supplementary Note 2. Screening effect of metals and dielectrics

Our device topology consists of a SiO_2 layer sandwiched between a metal gate and silicon substrate, with the donor embedded in the substrate. In such a topol-

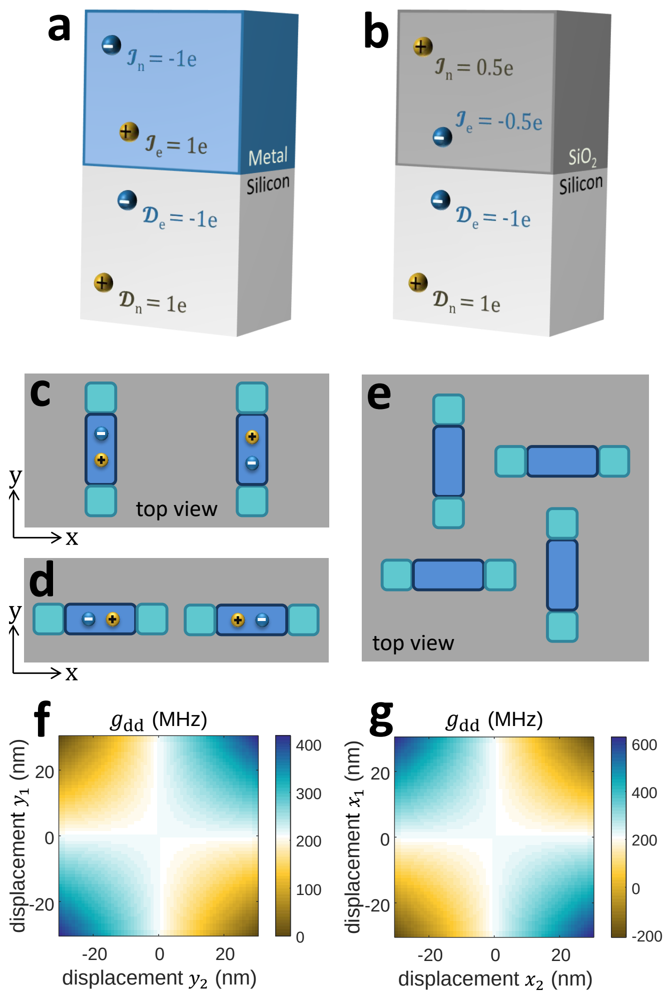

Supplementary Figure 2. **Screening and image charges.** Image ($\mathcal{I}_e$ and $\mathcal{I}_n$) charges of the donor electron ($\mathcal{D}_e$) and nucleus ($\mathcal{D}_n$) for silicon-metal (**a**) and silicon-oxide (**b**) interfaces. The magnitude and polarity of the image charges are given by Supplementary Eq. 1. Schematic top view of two interacting dipoles when the negative charges (blue spheres) are displaced in perpendicular (**c**) and parallel (**d**) direction to the inter-dipole separation. **e**, Top view of gate stack that tunes each qubit's V_t by displacing their interface states perpendicularly to their nearest neighbor displacement, leaving g_{dd} unchanged. Inter-dipole coupling g_{dd}, as predicted by Supplementary Eq. 4, for the orientation shown in **c** (**f**) and **d** (**g**), for $r = 200$ nm, $d_1 = d_2 = 10$ nm and $Q = -0.5$.

ogy, the image charges of the donor electron and nucleus will be located above the donor, thereby creating an additional vertical dipole. In this section, we quantify the variation of the dipolar coupling g_{dd} due to the electric field from the additional dipole, and arrive to the conclusion that g_{dd} will most likely be enhanced.

The magnitude and polarity of the image charges depend on the details of the nanostructure, such as the donor depth and thickness of the oxide. We first analyze two extreme scenarios considering image charges at (i) silicon-metal and (ii) silicon-oxide interfaces. For a source donor electron (or nuclear) charge $\mathcal{D}_{e(n)}$, in silicon, the image charge $\mathcal{I}_{e(n)}$ in the interface material is given by[3]

$$\mathcal{I}_{e(n)} = Q\ \mathcal{D}_{e(n)}, \tag{1}$$

$$Q = \frac{\epsilon_{Si} - \epsilon_I}{\epsilon_{Si} + \epsilon_I}, \tag{2}$$

where $\epsilon_{Si} = 11.7$ is the dielectric constant of silicon, $\epsilon_I = 3.9$ and ∞ for oxide and metal interfaces respectively. Supplementay figures 2a,b show the magnitude and polarity of the image charges for both types of interfaces. For simplicity, we assume in Supplementary Fig. 2 and Eq. 1 that the donor electron as well as its image are point charges. Given that the separation between the two donors is at least 180 nm (more than hundred times the Bohr radius of the donor electron), the above assumption is valid when calculating their dipolar interaction.

We first consider the electric dipole to be vertical. For the silicon-metal interface in Supplementary Fig. 2a, $Q = -1$ and therefore the image charges have the opposite sign and same magnitude as the source charges. As a result, the total electric field E_{dip} from each donor will be enhanced by a factor of 2. This improves the electric dipole coupling g_{dd} between the two donors by a factor of 4. On the contrary, for the silicon-oxide interface in Supplementary Fig. 2b, the image charges have the same sign and reduced magnitude ($Q = 0.5$) as the source charges, which decreases E_{dip} by half and therefore g_{dd} to a quarter of its bare value.

For a real device, which typically contains a few metal gates on top of a ~ 8 nm thick SiO_2, it is difficult to make a precise estimate of the extra electric field from image charges. Rahman *et. al.*[3] assumed that a combination of metallic and oxide screening effects yields $Q = -0.5$, corresponding to an improvement in the magnitude of the electric dipole by $\approx 50\%$, which yields an improvement in g_{dd} by 125%. This means that, while building a real device, one would have to aim for slightly larger inter-donor separations than the ones presented in the main text.

Since the donor-interface tunnel coupling V_t has to be tuned to a precise value, the dipole will also have lateral components as shown on the insets of Fig. 2g. These components will also be affected by image charges. In the case of a metallic interface, Supplementary Fig. 2a, the lateral image dipole has opposite direction as the original one, and therefore the total lateral component will be completely screened. On the other hand, for the SiO_2 interface, Supplementary Fig. 2b, the lateral component will be enhanced by 50%. Finally, for our assumed real structure ($Q = -0.5$), the lateral dipole will decrease to half its original value.

In total, the dipole size and orientation, including

screening, will be:

$$\mathbf{D}_i = \mathbf{d}_i + Q \times (d_{i,x}, d_{i,y}, -d_{i,z}), \quad (3)$$

where $\mathbf{d}_i$ refers to the bare dipole, with x, y and z components $d_{i,x}$, $d_{i,y}$ and $d_{i,z}$, respectively.

As a first consequence of image charges, the uncertainty in the total electric dipole of a donor-interface state is minimal, even when displacing the interface wavefunction laterally to tune up V_t. Indeed, at $V_t \approx 10$ GHz, the total dipole size of Supplementary Fig. 1 ($d_z = 11$ nm) is $(1+0.5)d_z = 16.5$ nm, while the one of Fig. 2g ($d_z = 5$ nm, $d_x = 25$ nm) is $\sqrt{[(1+0.5)d_z]^2 + [(1-0.5)d_x]^2} = 14.6$ nm. This is important for qubit reproducibility over a large scale processor.

And second, the dipole-dipole interaction term, Eq. 17, has to be modified to[8]:

$$g_{\mathrm{dd}} = \frac{e^2}{16\pi\varepsilon_0\varepsilon_r h} \frac{\mathbf{D}_1 \cdot \mathbf{D}_2 - 3(\mathbf{D}_1 \cdot \mathbf{r})(\mathbf{D}_2 \cdot \mathbf{r})/r^2}{r^3}, \quad (4)$$

which includes image charges and angular dependencies. Note that we neglect the interaction of a dipole with its own charge since it does not produce inter-donor coupling.

Laterally displacing the interface charge is, in general, necessary for the purpose of tuning the donor-interface tunnel coupling V_t. The same displacement, however, also alters the total electric dipole and can therefore affect the dipole-dipole coupling g_{dd} between neighboring qubits. We first consider the case in which the displacements are perpendicular to the separation between dipoles, Supplementary Fig. 2c. The g_{dd} dependence on y_1 and y_2 is plotted in Supplementary Fig. 2f, for maximum displacements of 30 nm (enough to tune V_t by two orders of magnitude – see Fig 2g). It shows that, provided that the interface states are displaced along the same direction, g_{dd} only varies by a factor of two. For completeness, we also analyze the case in which the interface states are displaced in the same direction as the inter-donor separation (Fig. 2d). As can be seen in the plot in Fig. 2g, g_{dd} varies by a factor of three if the interface states are displaced in opposite directions. Finally, the variation in g_{dd} can be reduced even further by fabricating the gate stack in such a way that the charges in neighboring qubits are displaced in perpendicular directions, as in Supplementary Fig. 2e. In this way, from Supplementary Eq. 4, the only dipole terms contributing to the coupling are the vertical ones, and therefore g_{dd} is unchanged (to first order) while tuning V_t.

Supplementary Note 3. Charge and gate noise

In the main manuscript, we have presented estimates of dephasing rates and gate errors extracted from models where we assume a quasi-static (*i.e.* with a spectral weight centered at frequencies smaller than the qubit resonance and the Rabi frequency) electric field noise acting on the qubits. Here we explain why this assumption, and the r.m.s. value of 100 V m^{-1} for the noise, is justified for silicon nanoelectronic devices.

The Si/SiO_2 interface is known to contain a number of defects and electron traps, which can generate charge noise and therefore degrade the operation of qubits sensitive to electric fields. Some experimental studies have extracted the trap density, in the middle of the silicon band gap, for the MOS devices we consider here[9]. It is known, experimentally and theoretically, that these charge fluctuators yield a $1/\nu$ frequency dependence of the noise spectral density[10]. These models capture the averaged collective effect of many charge fluctuators on the qubit operation. In specific cases, one can occasionally encounter individual charge traps or fluctuators whose effect is more drastic than that of an overall $1/\nu$ noise. However, it is usually possible experimentally to tune the electrostatic landscape of a nanoscale device in such a way that the individual trap is frozen, *i.e.* does not change its charge state while the qubit is operated. This results in a static shift in the local electric field that can be compensated with other gate voltages. In very rare occasions, a charge trap cannot be frozen while placing the qubit at its optimal operation point. In that case, the qubit will have to be considered faulty, and excluded from participating in the operations of the quantum processor.

In the general case where charge noise can be considered an average collective effect, it can be thought of as a quasi-static drift of the qubit electrostatic environment. Indeed, since individual gates take less than a microsecond, the qubit environment is usually static within a single gate, but fluctuates in between gates. This is exactly the noise model we assumed. Experimentally, average quasi-static charge detuning noises around 1-9 μeV are typically found in a range of semiconductor nanodevices, including SiGe[11–13], AlGaAs[14] and Si/SiO_2[13,15]. In particular, MOS structures where found recently[13] to have a charge noise spectrum similar to SiGe devices, around $(0.5\ \mu\mathrm{eV})^2/\nu$. Integrating over a quasi-static bandwidth relevant to experimental time-scales, say between 1 Hz and 1 MHz, yields 1.7 μeV noise. In our simulations, given that the distance between the donor and interface sites is $\sim$ 10-30 nm, a noise field of 100 V m^{-1} would correspond to 1-3 μeV charge detuning noise, therefore consistent with measured values.

In the main text, we have considered this quasi-static noise to be in the z-direction. Note that, when moving the interface wavefunction laterally to tune V_t, the electric dipole acquires some horizontal component – see Fig. 2g and Supplementary Note 2. In this case, the detuning noise is caused by the noise component along the donor-interface states direction. At the same time, horizontal noise will also have an effect, albeit minimal, in gate performance. For the parameters at which $V_t \approx 10$ GHz in Fig. 2g, 10 μV r.m.s. lateral noise

would cause less than 0.01% uncertainty in the dipole size, therefore causing negligible gate errors. The same noise causes less than 1% uncertainty in $\delta_{\rm so}$ (and therefore in gate time), which translates into maximum 10^{-4} errors due to gate time jitter, and maximum $\sim 10^4$ Hz extra dephasing due to dispersive shifts (Eq. 9).

Another source of electric field noise can be the thermal and electrical noise produced by the metallic gates on top of the qubits, and the room-temperature instruments they connect to. An $R = 50\ \Omega$ resistor at room temperature produces Johnson-Nyquist noise with an r.m.s voltage $\sqrt{4k_BTR\Delta\nu}$. Therefore a quasi-static bandwidth $\Delta\nu \sim 10^6$ Hz produces $\sim 1\ \mu$V voltage noise, which is equivalent to $E_{z,\rm rms}^{\rm noise} \sim 10$ V m^{-1}, or errors $< 10^{-5}$ (Fig. 4g). Furthermore, because of the very low powers required by the electrically-driven 1-qubit gates and adiabatic shuttling, it is possible to insert abundant low-temperature attenuation along the high-frequency lines, and therefore the relevant temperature for the Johnson-Nyquist noise is well below room temperature. On the other hand, being close to a metallic interface, our qubit will be subject to evanescent wave Johnson noise (EWJN) due to vacuum and thermal fluctuations. Assuming the qubit is $z = 15$ nm under aluminum gates at $T = 100$ mK ($\sigma = 1.4 \times 10^8$ S m^{-1} conductivity[16]), a quasi-static bandwidth $\Delta\nu \approx 10^6$ Hz produces[17] $\sqrt{k_BT\Delta\nu/(2z^3\sigma)} \sim 0.04$ V m^{-1} r.m.s. electric field noise, therefore negligible. We conclude that the main source of quasi-static noise will be charge noise with a typical $1/\nu$ spectrum. Consistently with recently measured for Si/SiO$_2$ interfaces[13], we assume the power spectral density to be $S_{\rm c}(\omega) \approx 10^4/(6\omega)$, in units of V^2 m^{-2} rad^{-1} s.

So far we have only considered quasi-static noise. The presence of some residual amount of high-frequency noise could possibly lead to errors while performing quantum operations. Below we discuss these high-frequency sources, finding that they will cause much smaller errors compared to quasi-static noise.

In general, a driven qubit Rabi-oscillates with a decay envelope function given by[18] $\zeta(t)\exp(-\Gamma_R t)$, where $\zeta(t)$ represents decay due to quasi-static detuning noise and Γ_R the exponential Rabi decay rate, which combines the qubit relaxation rate, Γ_1, the inverse of the gate time jitter due to quasi-static noise, Γ_1^{Δ}, the inverse of the gate time jitter due to noise at the drive frequency, Γ_1^{ν}, (the last three yield $T_{2\rho}$ in the dressed qubit picture[19]) and the decay rate due to detuning noise at the Rabi frequency, Γ_Ω (which equals the inverse of $T_{1\rho}$ in the dressed qubit picture[19,20]).

The effects of $\zeta(t)$, Γ_1 and Γ_1^{Δ} have already been discussed extensively in this manuscript, with corresponding error levels below 10^{-3}. We now focus on errors due to high-frequency noise sources, corresponding to decay rates Γ_1^{ν} and Γ_Ω.

Vertical (thus parallel to the driving field $E_{\rm ac}$) noise at the qubit resonance frequency ($\sim 10^{10}$ Hz) would cause transitions between the qubit eigenstates – essentially a spurious excitation/relaxation process driven by noise – at a rate Γ_1^{ν}. This noise can be caused *e.g.* by charges fluctuating in resonance with the qubit or by voltage noise at the metallic gates. This includes vacuum fluctuations, especially since the qubit frequency is generally higher than the corresponding device temperature. Also, during gate operations, the portion of the noise spectrum around the qubit frequency can add incoherently to the external resonant drive, causing the gate time to fluctuate. For the flip-flop qubit, the Rabi decay rate is given by $\Gamma_1^{\nu} = (\pi/2)(\mu_e^{\rm ff}/\hbar)^2 S(2\pi\epsilon_{\rm ff})$, where $\mu_e^{\rm ff} = ed\langle g_{\rm so}/\delta_{\rm so}\rangle$ is the average flip-flop qubit electric dipole moment and $S(2\pi\epsilon_{\rm ff})$ is the noise power spectral density at the qubit angular frequency (in units of V^2 m^{-2} rad^{-1} s). Note that this flip-flop electric dipole is much smaller than the charge dipole, which in turn makes it less susceptible to electrical noise. This happens because, in our gate scheme, the charge dipole is only used as a second-order enabler, and therefore charge excitation is greatly minimized (Figs. 3a, 4c and 6a). In case of charge noise, $S_{\rm c}(\omega) = 10^4/(6\omega)$, which gives $\Gamma_1^{\nu} \sim 10^4$ Hz. This implies $\pi/2$ x-gate errors $\sim 10^{-4}$. There could also be vacuum fluctuations of charge traps, which could also generate errors due to relaxation. We do not know of any experimental measurement of such a noise for semiconductor nanostructures. For superconducting charge qubits, it has been found that charge noise increases linearly at frequencies beyond the thermal bath[21], which has been explained in terms of two-level coherent charge fluctuators[22]. If a similar phenomenon afflicts our qubits, those quantum fluctuations will play an important role beyond ~ 2 GHz (100 mK), implying that, at 10 GHz, relaxation can be up to 25 times faster. This can increase relaxation error rates to $\sim 10^{-3}$. In case of Johnson-Nyquist noise, $S_{\rm JN}(\omega) = 2\times10^{14}R\hbar\omega\pi^{-1}(e^{\hbar\omega/k_BT}-1)^{-1}$ (where we have used $\partial E_z/\partial V = 10^7$ m^{-1}, typical in MOS nanostructures). Because of the very low powers required by the electrically-driven 1-qubit gates (< 1 pW), it is possible to insert abundant low-temperature attenuation along the high-frequency lines, insuring that the gates are well thermalized, and the noise of the room-temperature electronics greatly attenuated. A noise temperature $T = 100$ mK would give $\Gamma_1^{\nu} < 10^4$ Hz, and therefore error rates $< 10^{-4}$. Finally, in case of EWJN at $T = 100$ mK, the 10^{10} Hz part of the spectrum is[17,23] $S_{\rm EW}(\omega) \approx \hbar\omega/(4\pi z^3\sigma)$. This would give $\Gamma_1^{\nu} < 10^4$ Hz, therefore again error rates $< 10^{-4}$.

Noise at the Rabi frequency ($\Omega_R > 10^7$ Hz) causes decay in the Rabi oscillations at a rate Γ_Ω. This type of noise feeds into the driven qubit via fluctuations in the detuning between drive frequency and the qubit precession frequency. The decay rate of the flip-flop qubit is given by $\Gamma_\Omega = (\pi/2)(2\pi\sum_{i=x,y,z}\partial\epsilon_{\rm ff}/\partial E_i)^2 S(\Omega_R)$. At the low-error operation region of Fig. 4f, $\partial\epsilon_{\rm ff}/\partial E_z \sim 10^3$ Hz V^{-1} m and $\partial\epsilon_{\rm ff}/\partial E_{x,y} \sim 10^2$ Hz V^{-1} m (from Fig. 2g). $1/\nu$ charge noise gives $\Gamma_\Omega < 10^4$ Hz, implying $< 10^{-4}$ errors. Johnson-Nyquist noise from room temperature gives $\Gamma_\Omega = 3 \times 10^2$ Hz, whereas EWJN

at 100 mK gives $\Gamma_\Omega = 2 \times 10^1$ Hz, therefore producing $< 10^{-5}$ and $< 10^{-6}$ errors, respectively.

We conclude that the sources of error treated in the main text, namely quasi-static E_z noise and charge-phonon relaxation, are the most deleterious ones for flip-flop qubits. Therefore our analysis is sufficient to provide a reliable estimate of dephasing and gate errors. Indeed, low-frequency noise was found to be the most deleterious one in a hybrid donor-dot qubit in a silicon MOS device[15]. Finally, note that we do not assume any type of dynamical noise correction or cancellation to be applied, and therefore our calculations are a worst-case scenario.

Supplementary References

1. Kohn, W. & Luttinger, J. M. Theory of donor states in silicon. *Phys. Rev.* **98**, 915–922 (1955).
2. Saraiva, A. L., Calderón, M. J., Hu, X., Das Sarma, S. & Koiller, B. Physical mechanisms of interface-mediated intervalley coupling in Si. *Phys. Rev. B* **80**, 081305 (2009).
3. Rahman, R. *et al.* Orbital stark effect and quantum confinement transition of donors in silicon. *Phys. Rev. B* **80**, 165314 (2009).
4. Calderón, M. J., Saraiva, A., Koiller, B. & Das Sarma, S. Quantum control and manipulation of donor electrons in Si-based quantum computing. *J. Appl. Phys.* **105**, 122410 (2009).
5. Klimeck, G. *et al.* Atomistic simulation of realistically sized nanodevices using NEMO 3-D – part I: Models and benchmarks. *Electron Devices, IEEE Transactions on* **54**, 2079–2089 (2007).
6. Klimeck, G. *et al.* Atomistic simulation of realistically sized nanodevices using NEMO 3-D – part II: Applications. *Electron Devices, IEEE Transactions on* **54**, 2090–2099 (2007).
7. Yang, C. H., Rossi, A., Ruskov, R., Lai, N. S., Mohiyaddin, F. A., Lee, S., Tahan, C., Klimeck, G., Morello, A. & Dzurak, A. S. Spin-valley lifetimes in a silicon quantum dot with tunable valley splitting. *Nature Commun.* **4**, 2069 (2013).
8. Ravets, S., Labuhn, H., Barredo, D., Beguin, L., Lahaye, T. & Browaeys, A. Coherent dipole-dipole coupling between two single Rydberg atoms at an electrically-tuned Rörster resonance. *Nature Phys.* **10**, 914–917 (2014).
9. Johnson, B. C., McCallum, J. C., Willems van Beveren, L. H., Beguin, L. & Gauja, E. Deep level transient spectroscopy study for the development of ion-implanted silicon field-effect transistors for spin-dependent transport. *Thin Solid Films* **518**, 2524 (2010).
10. Paladino, E., Galperin, Y. M., Falci, G. & Altshuler, B. L. $1/f$ noise: Implications for solid-state quantum information. *Rev. Mod. Phys.* **86**, 361–418 (2014).
11. Kim, D. *et al.* High-fidelity resonant gating of a silicon-based quantum dot hybrid qubit. *npj Quantum Information* **1**, 15004 (2015).
12. Thorgrimsson, B. *et al.* Extending the coherence of a quantum dot hybrid qubit. Preprint at `http://arxiv.org/abs/1611.04945` (2016).
13. Freeman, B. M., Schoenfield, J. S., Jiang, H. Comparison of low frequency charge noise in identically patterned Si/SiO_2 and Si/SiGe quantum dots. *Appl. Phys. Lett.* 108, 253108 (2016).
14. Dial, O. E. *et al.* Charge noise spectroscopy using coherent exchange oscillations in a singlet-triplet qubit. *Phys. Rev. Lett.* **110**, 146804 (2013).
15. Harvey-Collard, P. *et al.* Coherent coupling between a quantum dot and a donor in silicon. Preprint at `http://arxiv.org/abs/1512.01606` (2015).
16. Dehollain, J. P., Pla, J. J., Siew, E., Tan, K. Y., Dzurak, A. S. & Morello, A. Nanoscale broadband transmission lines for spin qubit control. *Nanotechnology* **24**, 015202 (2013).
17. Henkel, C., Pötting, S. & Wilkens, M. Loss and heating of particles in small and noisy traps. *App. Phys. B* **69**, 379–387 (1999).
18. Bylander, J. *et al.* Noise spectroscopy through dynamical decoupling with a superconducting flux qubit. *Nature Phys.* **7**, 565–570 (2011).
19. Laucht, A. *et al.* A dressed spin qubit in silicon. *Nature Nanotechnol.* **12**, 61–66 (2017).
20. Yan, F. *et al.* Rotating-frame relaxation as a noise spectrum analyser of a superconducting qubit undergoing driven evolution. *Nature Commun.* **4**, 2337 (2011).
21. Astafiev, O. *et al.* Quantum noise in the Josephson charge qubit. *Phys. Rev. Lett.* **93**, 267007 (2004).
22. Shnirman, A. *et al.* Low- and high-frequency noise from coherent two-Level systems. *Phys. Rev. Lett.* **94**, 127002 (2005).
23. Poudel, A., Langsjoen, L. S., Vavilov, M. G. & Joynt, R. Relaxation in quantum dots due to evanescent-wave Johnson noise. *Phys. Rev. B* **87**, 045301 (2013).
